# Sex differences in the response to high pulse pressure in APOE4 mice

**DOI:** 10.1002/alz.091925

**Published:** 2025-01-03

**Authors:** Skylyn J Ferguson, Lazar N Isakharov, Ashley E Walker

**Affiliations:** ^1^ University of Oregon, Eugene, OR USA

## Abstract

**Background:**

Elevated arterial pulse pressure (PP) is associated with cognitive decline and Alzheimer’s disease (AD). High PP damages the brain vasculature by causing endothelial cell dysfunction. Stiffer cerebral arteries have an impaired ability to dampen PP, which transmits the pulsatility further into the microvasculature, where it can damage brain tissue. At the same time, the APOE4 genotype is associated with cerebrovascular dysfunction; however, it is unknown if elevated PP amplifies this risk. Furthermore, female APOE4 carriers are at a higher risk for developing AD than males. Thus, we hypothesized that female APOE4 mice would be more vulnerable to high PP and have greater cerebral artery stiffness and cognitive impairments compared with male mice.

**Method:**

In male and female mice homozygous for APOE4 and humanized Aβ1‐42 (APOE4/hAβ, n = 26, 6 months), we assessed ex vivo endothelium‐dependent vasodilation by the dose‐responses to acetylcholine in isolated posterior cerebral arteries (PCAs) following exposure to static pressure (50 mmHg), low PP (50‐75 mmHg), or high PP (37.5‐87.5 mmHg). PCA endothelium‐independent vasodilation was measured by the response to sodium nitroprusside, and stiffness was measured during the high PP condition. We assessed cognition by the Morris Water Maze.

**Result:**

Under static pressure, PCA endothelium‐dependent vasodilation was similar between male and female mice (p = 0.13). In females, PCA endothelium‐dependent vasodilation was similar between static and low PP (p = 0.37), while exposure to high PP resulted in a 38% decline in endothelium‐dependent vasodilation (p = 0.004 vs. static). In contrast, PCA endothelium‐dependent vasodilation in male mice was similar between static and PP conditions (p>0.05). Endothelium‐independent vasodilation did not differ between sexes (p>0.05). Female mice also exhibited a higher PCA β‐stiffness index than male mice (p = 0.03). Additionally, during the Morris Water Maze probe trial, females crossed the platform area significantly fewer times than males (p = 0.048).

**Conclusion:**

Our data indicate that high PP is detrimental to cerebral artery endothelial function in female, but not male, APOE4/hAβ mice. Additionally, female APOE4/hAβ mice have stiffer cerebral arteries and poorer spatial memory than male APOE4/hAβ mice. Our findings suggest that sex may influence the interactive effect of APOE4 and PP on cerebrovascular and AD‐related outcomes.

